# Diagnosing Extrusion Process Based on Displacement Signal and Simple Decision Tree Classifier

**DOI:** 10.3390/s22010379

**Published:** 2022-01-05

**Authors:** Grzegorz Piecuch, Rafał Żyła

**Affiliations:** 1 Department of Computer and Control Engineering, Rzeszow University of Technology, 35-959 Rzeszów, Poland; 2 EDOCS Systems Sp. z o.o., 35-105 Rzeszów, Poland; r.zyla@edocssystems.com

**Keywords:** extrusion process, displacement signal, decision tree, anomaly detection, polynomial approximation, process diagnosing

## Abstract

The article presents an extensive analysis of the literature related to the diagnosis of the extrusion process and proposes a new, unique method. This method is based on the observation of the punch displacement signal in relation to the die, and then approximation of this signal using a polynomial. It is difficult to find in the literature even an attempt to solve the problem of diagnosing the extrusion process by means of a simple distance measurement. The dominant feature is the use of strain gauges, force sensors or even accelerometers. However, the authors managed to use the displacement signal, and it was considered a key element of the method presented in the article. The aim of the authors was to propose an effective method, simple to implement and not requiring high computing power, with the possibility of acting and making decisions in real time. At the input of the classifier, authors provided the determined polynomial coefficients and the SSE (Sum of Squared Errors) value. Based on the SSE values only, the decision tree algorithm performed anomaly detection with an accuracy of 98.36%. With regard to the duration of the experiment (single extrusion process), the decision was made after 0.44 s, which is on average 26.7% of the extrusion experiment duration. The article describes in detail the method and the results achieved.

## 1. Introduction

In line with the idea of industry 4.0, one should strive for increasing digitization, diagnosing processes, predicting failures and reducing the number of defects, etc. This idea is implemented to a different extent in various industries—one of such industries where more and more emphasis is placed on the use of new technologies in production are the automotive and aviation industries. The quality of the produced details (product or semi-finished product) and minimizing errors is a key issue. In these industries, we deal mainly with machining processes (milling, turning, grinding), casting, thermal processes, etc. The problems of diagnosing machines and industrial processes have been known for decades. Now, however, thanks to the development of IoT and the understanding of the needs behind the definition of Industry 4.0, specialized platforms for the diagnosis and supervision of processes and machines have been created [1]. Various areas of knowledge are developed, e.g., data integration from multiple sources (e.g., sensors) [2] and effective feature extraction methods [3], etc. One of the processes that can be diagnosed is the extrusion process. This article presents a very simple way to diagnose this process. It is not always necessary to use sophisticated machine learning methods or expensive sensors to solve the problem, as evidenced by the authors’ approach to diagnosing the extrusion process. The authors did not find solutions in the literature that use displacement sensor for the extrusion process diagnosis. Due to this fact, the authors present a novelty diagnostic method for extrusion process, with displacement signal as the unique value, while the algorithm based on polynomial approximation complements the proposed method. The main aim of this publication was to utilize a displacement sensor in a new and real application.

## 2. Extrusion Process—Analysis

Extrusion is a difficult process, closely related to many fields of knowledge, including mechanics, materials science and others. In the case of extrusion metal details, coefficients such as elastic and plastic deformation of the material, die lubrication, as well as static and dynamic properties of presses should be taken into account. Changing even one process parameter may cause an anomaly, and consequently produce a defective detail [4]. The process is non-linear [4], and has over 40 process variables related to the state of the die, material characteristics or press configuration parameters [5]. In the past, the vibration signal, which is typical for the milling process, was analyzed [6], as well as the temperature on selected tool surfaces, operation time of process [7] and punch feed rate [8]. Despite this, the pressing force is considered to be the most important process variable in extrusion as the most direct and authoritative [9]. According to [5], the compression force signal is useful in predicting the quality of the extruded detail, since any anomaly detected in the course of this type of signal reflects a change in process conditions that may affect the manufacturing process.

In the research described in [7], a decision support system based on artificial neural networks (ANN) was proposed, the task of which was to analyze and forecast the durability of forging tools used in the process of extruding the cover of an element sealing the drive shaft in trucks. To develop the system, input signals were used, such as: the number of details made; pressure force; temperature on selected tool surfaces (image from a thermal imaging camera); and operation time.

The authors [8] tested three machine learning methods (ANN, OC-SVM: One-Class Support Vector Machine, IF—Isolation Forest) to detect anomalies in the extrusion process. The input data were the signals of the force and the feed rate of the punch, although the pressure in the cylinders and the temperature of the hydraulic oil were also measured. The ANN network showed the best anomaly detection accuracy (100%), followed by the Isolation Forest classifier (99.5%) and the OC-SVM (99.4%). However, it was indicated that the ANN method took longer to learn, and that it was more difficult to implement than the other tested methods.

In [4], it was indicated that in the case of the extrusion process, the most commonly used sensors are extensometers, force sensors and accelerometers. The advantages of using vibration sensors were listed: ease of data acquisition, independence from environmental variables, but also a large reflection of dynamic processes in the signal. The disadvantage was the sensitivity, which in the case of a low amplitude signal and the presence of interference, does not always allow to extract features solely related to extrusion from the signal. The tests were carried out on a SN1-25 press with a nominal pressure of 25 tons of additional equipment extensometers, from which the signal was recorded with a sampling frequency of 1 kHz. The LVQ (Learning Vector Quantization) classifier was prepared, built on the empirical decomposition method and the Hilbert transform, which recognized three classes: good pass; bad pass; and too-thin material. The accuracy of classification on the test data was 96.7%. The same press was also used in the research [10], where the SVM (Support Vector Machine) algorithm was tested for six classes (accuracy above 96.5%), and in [11], where the analogous six classes were also tested by the classifier based on hidden Markov models (accuracy in the range of 82.5–91.67%).

In [12] force sensors integrated with the die was proposed (8 pcs). The simulation was carried out in the Ansys environment, and then the assumptions were verified in reality on a physical press. The proposed technique, according to the authors, can significantly contribute to the development of knowledge in cases where the extruded detail is characterized by a large contact area with the tool. This research was developed in [13] by increasing the number of force sensors, and thus more precisely interpolating the pressure distribution.

Force sensors were also used in [14], where a two-stage system for diagnosing the extrusion process was proposed. First, the signals from the force sensors were analyzed, then the finished detail was placed in a special cabin equipped with two cameras, the image of which was automatically analyzed for damage. The system was based on the fuzzy logic algorithm. The accuracy of the system, depending on the type of damage to the detail, oscillated between 89–92%.

In [15], it is noticed that many sensors and methods used to improve the quality of extruded details, as well as the influence of the working condition of tools (e.g., stamps) on it, have already been tested for the extrusion process. It was decided to investigate the correlation of the acoustic signal in the extrusion process with the condition of the tool, which was finally confirmed. Data was collected from three microphones and a position sensor with a sampling rate of 12 kHz. The research work was extended and described in [16] using ultrasonic sensors. The signals were analyzed in the time domain, as well as in the time-frequency domain. RMS and peak ratios showed a strong correlation with the maximum surface depth of the embossed parts. The methods of WPT (Wavelet Packet Transform), STFT (Short-Time Fourier Transform) and HHT (Hilbert-Huang Transform) were also compared, and it was found that in the case of used matrices the signal was so unsteady that only the HHT method gave positive results.

Acoustic emission sensors were also used in [17], where the high potential of this type of signals was confirmed in diagnostic methods of the extrusion process. However, the authors pointed out that the signals will not work in the case of forming details from plastic materials, due to the fact that the cracks in the material during stretching generate a signal with too small amplitude.

As can be seen from the above analysis, the methods used to diagnose the extrusion process at the moment use expensive solutions (accelerometers), difficult to assemble (extensometers) and complicated to implement.

The authors of this publication have noticed that the punch displacement itself contains sufficient data for effective process diagnostics. The results of our research are presented in the following chapters.

## 3. Materials and Methods

**Figure 1:**
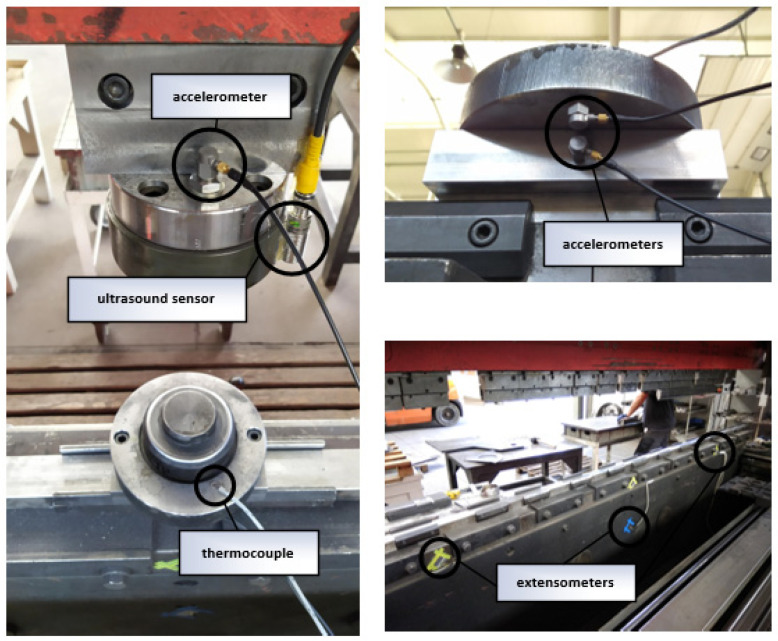
Arrangement of sensors on the machine.

The extrusion process was recorded on an Amada HFP 170-4 L hydraulic press, on which details for the automotive industry were extruded. An IPC Beckhoff C6015 industrial computer with modules EL3632, EL3312, EL3702 and EL3064, to which accelerometers (3 pcs), extensometers (3 pcs) and a displacement sensor were connected (Balluff BUS0024). This article describes a process diagnostic method based only on the signal from the displacement sensor. Data were recorded with a sampling rate of 0.5 kHz for the displacement sensor, 1 kHz for extensometers and 25 kHz for accelerometers. The arrangement of the sensors is shown in Figure 1.

**Figure 2:**
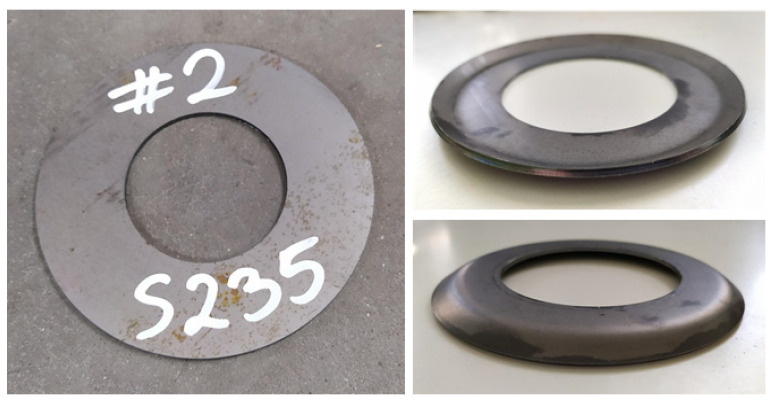
Detail before (**left**) and after extrusion (**right**).

The extruded detail was a ring with an outer diameter of 115 mm and an inner diameter of 69 mm. The ring was made of the materials S235 and PA11, and had a different thickness of 2 or 4 mm. Its shape before and after extrusion is shown in Figure 2.

**Figure 3:**
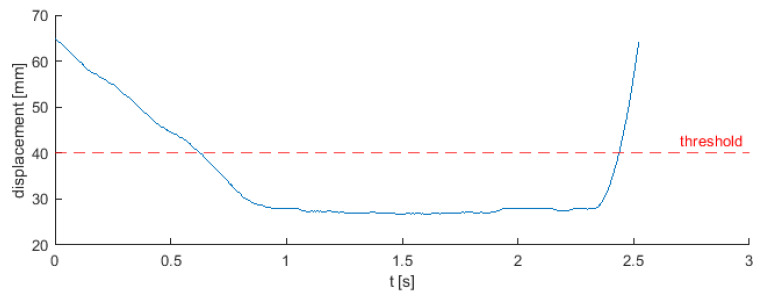
Example displacement signal from the entire process and 40 mm threshold.

A special device was mounted in the press, which was a form for the extruded detail. It consisted of a die and a punch on which an ultrasonic displacement sensor was mounted. During the process, the punch lowers towards the die, extrudes the detail, and then moves back to the base position after extrusion. In this case, the thicker detail was 4 mm, but after extrusion it was 8 mm (the distance between the lowest and highest points of the sample). The press also pressed 6 mm thick samples, which after extrusion had a thickness of 10 mm (during these works, 6 mm details were not registered). Taking into account the location of the sensor installation, a universal initial distance 40 mm between the punch and the matrix was selected, which was also the threshold initiating the experiment and thus the data recording. Any measurement above this value was automatically rejected. This distance had to take into account the thickness of the sample after extrusion and a certain dimension and measurement tolerance. The lowest measured point of all the recorded signals was 26.06 mm, which means that the measuring range was less than 14 mm. An exemplary displacement signal after performing the above procedure looks as shown in Figure 3. Database contain a 62 registered signal of displacement, where each instance was represented by raw data from the sensor and timestamp.

As shown in Figure 3, the signal from the distance sensor is slowly changing, and perfectly reflects the movement of the punch depending on the phase of the process. Since diagnosing method has to work in real-time rigor, authors decided to simplify the algorithm and feature extraction process as much as possible. It was also decided to maximize the usefulness of the algorithm by adapting it to the fact that it could report potential anomalies in real time at the very beginning of the process, so that it could be interrupted and consequently save time. Thanks to these boundary conditions, after observation of the collected data, the first 400 samples were separated from each experiment, which corresponded to a duration of 0.8 s.

The four cases were recorded, where 18 from 62 instances is correct (data is unbalanced):

- Correct process: S235 (4 mm)—18 instances;
- Incorrect process: S235 (2 mm)—17 instances, PA11 (4 mm)—25 instances, no detail in the die—2 instances.

The starting level of the displacement signals was set at a distance of 40 mm between punch and die. The collected time series were normalized (each observation separately) to the range [0, 1], with the following Equation (1) [18]

(1) $$y_{\mathrm{norm}}=\frac{y-y_{\min}}{y_{\max}-y_{\min}}$$

where:

y_norm_—normalized value of displacement,
y—value of displacement in [mm].

**Figure 4:**
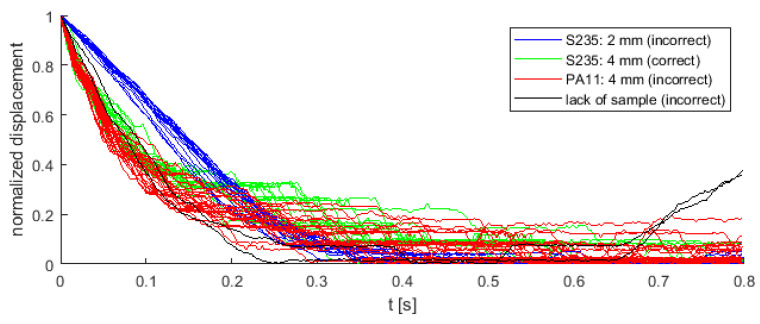
Sample normalized displacement signals from the extrusion process.

**Figure 5:**
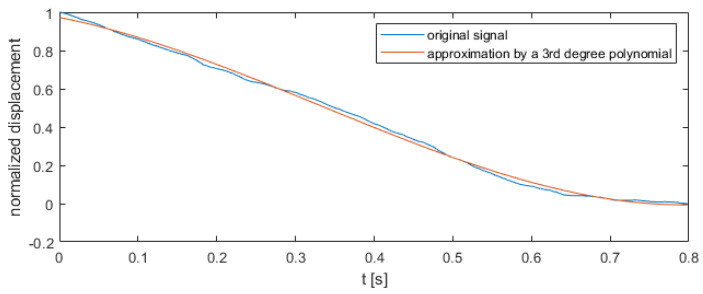
Original signal of displacement and approximation by 3rd degree polynomial.

As shown in Figure 4, the curve from all experiments were overlayed, except for the detail made of S235 material with a thickness of 2 mm. The remaining cases (including the correct process) overlay and are not linearly separable from each other. All details, regardless of the material and thickness, were extruded with the same pressing force (800 kN). In this paper, a decision tree was used to distinguish correct and incorrect extrusion process. The classifier was fed with polynomial coefficients fitted to displacement values with timestamps as explanatory variable. Function polyfit from Matlab was used to find these coefficients. Similar approach was used in classification of supernovae [19,20,21], where parameters of special functions fitted to light curves of supernovae were used as input feature vectors for classification methods (such as neural networks or support vector machines). An approach based on polynomial coefficients was chosen in order to describe the shape of every time series, which might be different in correct and incorrect class. Exemplary approximation of displacement signal by 3rd degree polynomial is shown in Figure 5.

For example, an approximated signal of experiment shown in Figure 5 was described by Equation (2)

0.0326x^3^ + 0.0281x^2^ − 0.3807x + 0.3994 (2)

where

x—timestamp [s].

**Figure 6:**
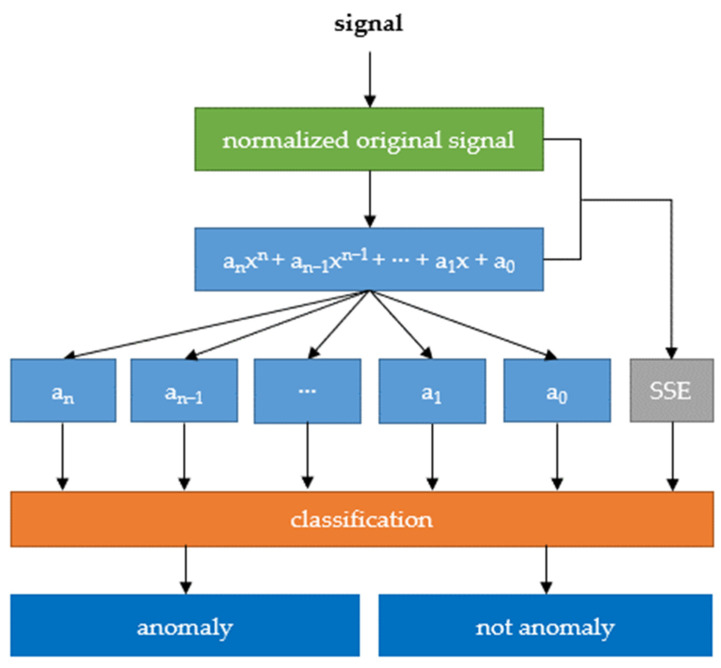
Schema of principle operation of the algorithm.

Additionally, to coefficients of fitted polynomials, a sum of squared errors (SSE) calculated between original and approximated signal was entered to the input of classifier. The idea of a classifier was presented as a schema on Figure 6.

## 4. Results

**Figure 7:**
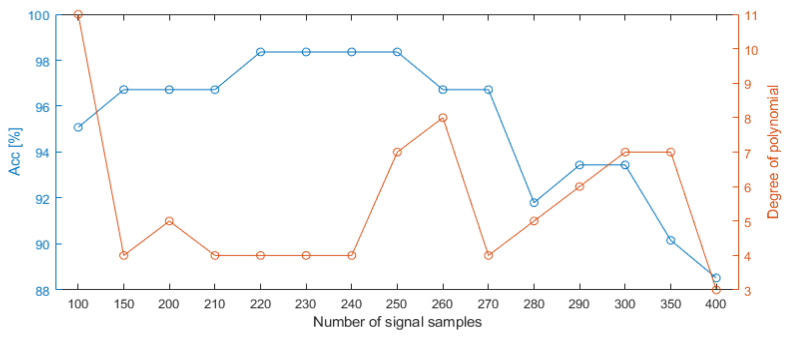
Classification accuracy in relation to the signal length and the minimum degree of the polynomial to achieve the indicated accuracy.

**Figure 8:**
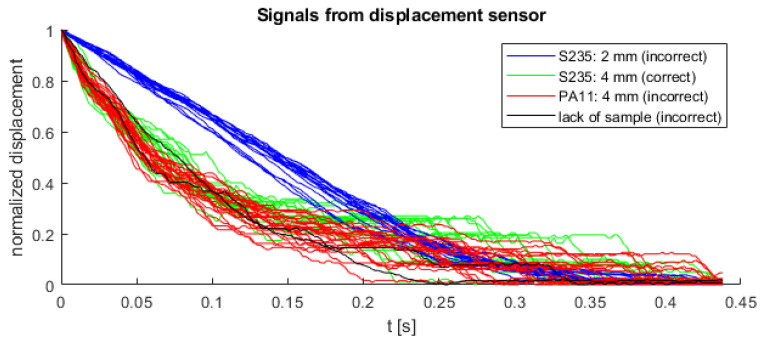
Normalized displacement signals from the extrusion process (first 220 samples).

All collected data were cross-validated 10 times to determine the set of training and test data. During the training process of the classifier, the optimization of hyperparameters (maximum number of splits and minimum leaf size) was performed by the grid search method. The structure of the classifier was built on the decision tree model (CART). The degree of polynomial was treated as a hyperparameter, and the optimal value was found through cross-validation. Finally, the confusion matrix for each degree of polynomial and number of samples was a result of sum a values of 10 classification results for each part of data. In order to find the appropriate degree of the polynomial, an automatic selection was carried out, consisting of determining the polynomial coefficients for the degree from 1 to 15, where each time these coefficients were entered at the input of decision tree classifier. The procedure was repeated also for a different number of signal samples in order to find the shortest possible signal without losing the classification quality. Attempts were made to test the classification for the learned classifier on the basis of a signal with a length of 100 to 400 samples, with the range of 200–300 with an interval of 10 (due to the observed higher classification quality indicators) and 100–200 and 300–400 with an interval of 50 samples. The analysis of the performed tests showed that with 220 samples of the measurement signal it is possible to achieve 98.36% classification accuracy with only the 4th degree of the polynomial and one predictor (SSE). Additionally, in this case, the depth of decision tree was 1, where root was marked as 0. Figure 7 shows the dependence of the classification accuracy on the number of signal samples and the minimum polynomial degree that allowed to achieve this accuracy. In the case that the same accuracy was obtained for different degrees of the polynomial with the same number of samples, the lowest degree was always selected. Likewise, when the same classification accuracy was obtained for a different number of samples, the smallest number of samples was always selected. Both conditions were assumed to translate into a shorter reaction time of the algorithm and lower computational complexity. The interpretation of the graph in Figure 7 comes down to finding the greatest amplitude between the degree of the polynomial and the accuracy, with the smallest number of signal samples. All collected and normalized displacement signals (62 experiments) for 0.44 s (220 samples) were presented in Figure 8.

**Figure 9:**
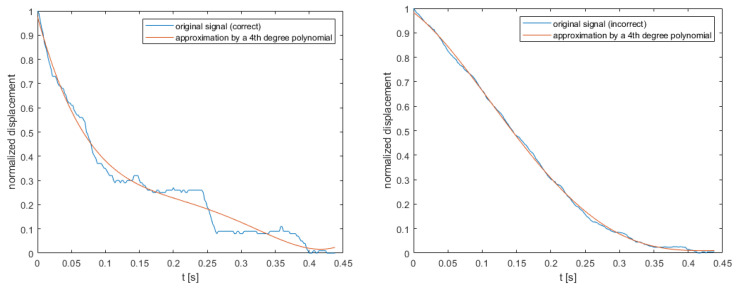
Original correct (on the **left**) and incorrect (on the **right**) signal of displacement and approximation by 4th degree polynomial.

For 220 samples, exemplary approximation of displacement signals by 4th degree polynomial is shown in Figure 9, and detailed classifier parameters (for each degree of polynomial) were presented in Table 1. Classifiers performance was measured with accuracy, along with four additional metrics that are robust to unbalance data [22]:

- Accuracy—$\mathrm{Acc}=\frac{\mathrm{TP}+\mathrm{TN}}{\mathrm{TP}+\mathrm{TN}+\mathrm{FN}+\mathrm{FP}}$
- Sensitivity—$\mathrm{Sen}=\frac{\mathrm{TP}}{\mathrm{TP}+\mathrm{FN}}$
- Specificity—$\mathrm{Spe}=\frac{\mathrm{TN}}{\mathrm{TN}+\mathrm{FP}}$
- Positive Predictive Value—$\mathrm{PPV}=\frac{\mathrm{TP}}{\mathrm{TP}+\mathrm{FP}}$
- False Alarm Rate—$\mathrm{FAR}=\frac{\mathrm{FN}}{\mathrm{FN}+\mathrm{TP}}$

where TP is number true positives, TN is number of true negatives, FN is number of false negatives and FP is number of false positives. In this case, an anomaly (incorrect state) is marked as positive.

Coefficients of fitted polynomial for correct signal from Figure 9 was presented below (3)

0.0362x^4^ − 0.0553x^3^ − 0.0093x^2^ − 0.1132x + 0.2109 (3)

where x—timestamp [s].

**Figure 10:**
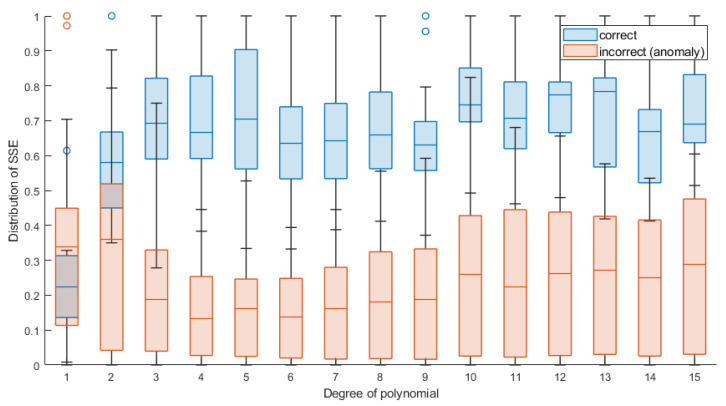
Distribution of SSE value (rescaled to 0–1 range) of each degree polynomials approximation of the displacement signals (for case of 220 samples).

Figure 10 shows per class normalized distribution of SSE value for each of polynomial degree. With regard to the classification results from Table 1, where the 4th degree polynomial was selected as the minimum degree to the best classification results, the SSE distribution shows that the first two degrees are characterized by a significantly greater error of approximation in relation to the next degrees. Additionally, for the first two degrees of the polynomial, the SSE distribution overlaps in both classes. Only from level 3 upwards it is noticeable that the SSE values are clearly separable, and this parameter is a very good discriminator in this case.

The proposed method was compared with other data classification algorithms, based on the same input data as for the selected best data set for the decision tree described above. Therefore, as input data, the coefficients of the 4th degree polynomial and the determined SSE value based on the signal from 220 samples were used. The following methods were tested: discriminant analysis (LDA—linear and QDA—quadratic); logistic regression (LR); naive Bayes classifier (NB); SVM (Support Vector Machine); kNN (k-nearest neighbor); multilayer perceptron (MLP); and Bagged Trees. With the exception of logistic regression, optimization of hyperparameters was performed for all other algorithms. The same cross-validation approach mentioned above was used to obtain results for classifiers presented below.

In Table 2, the classification results for individual algorithms were presented, sorted from highest to lowest accuracy. For some algorithms, different combinations of hyperparameters allowed to achieve the same classification result. A result of decision tree (SDT) algorithm was also presented in Table 2 for easier comparison (bold text).

Some of the tested classifiers achieve exactly the same results as the decision tree, though the decision tree with depth equal to 1 remains the easiest algorithm to implement in embedded systems. Due to this fact, a decision tree comes down to the one if-statement.

## 5. Discussion

As mentioned in the analysis of the literature in Section 2, diagnostics of the extrusion process are not easy, due to many variables influencing the process. The proposed use of the ultrasonic distance sensor seems non-obvious, but effective. The ultrasonic sensor can be replaced for example with an optical sensor with analog output. When selecting a sensor, it should be remembered that they often have a blind spot at the beginning of the measuring range, and therefore it is necessary to select an appropriate place of installation.

The implementation of the method proposed in the article was developed in Matlab using the polyfit function and the decision tree algorithm. It should be taken into account that both the determination of the coefficients of the polynomial and the preparation of the classification algorithm can be carried out using, for example, Python or microPython languages, which can be used for commercial purposes free of charge. Thus, the method can be implemented on a cheap embedded system such as the Raspberry Pi, which has sufficient computing power to operate the algorithm and make decisions in real time during the process of extruding a given detail.

Of course, one can consider deep learning algorithms (e.g., LSTM or 1D-CNN) or algorithms typically designed for the time series described e.g., in [23]. The authors, however, made it a goal to make the solution as relatively simple as possible. Unfortunately, deep learning methods often require a large amount of training data, the learning process is much longer than the exemplary decision tree, the selection of appropriate hyperparameters is troublesome, and they are also complicated to implement.

## 6. Conclusions

The proposed solution is unique; so far, no similar solution based only on the displacement signal has been found in the literature. The authors consider this to be the unique value and key element of the proposed method. In addition, the ultrasonic distance sensor is a cheap sensor, returning the signal in the typical ranges known from automation (e.g., 0–10 V, 4–20 mA, or via a communication protocol, e.g., IO-Link) and sampled with a relatively low frequency (0.5 kHz). The proposed decision tree algorithm is very simple and therefore easy to implement, and requires no computing power. Thanks to this, there is no need to use an expensive PLC controller, but it could successfully use a simple embedded system (e.g., Raspberry Pi) that operates and diagnoses the process in real time. In addition, due to the fact that only the initial part of the signal is used for diagnosis, the incorrect course of the process can be interrupted and thus save time, which can effectively contribute to the economic aspects of the production process. The average value of signal length was about 823 samples (corresponding to 1.646 s), and the only first 220 samples of signal was necessary to detect anomalies (98.36% accuracy) in the extrusion process (approx. 26.7% of length of signal).

## Figures and Tables

**Table 1:** Result of classification based on 220 samples of signal for decision tree classifier. LT—learning time, DDT—depth of decision tree.

	Degree of Polynomial														
	1	2	3	4	5	6	7	8	9	10	11	12	13	14	15
ACC [%]	79.03	75.81	90.32	98.39	90.32	98.39	98.39	93.55	91.94	87.1	88.71	87.1	90.32	95.16	90.32
SEN [%]	88.64	86.36	93.18	100	95.45	100	100	95.45	100	90.91	93.18	90.91	95.45	95.45	90.91
SPE [%]	55.56	50	83.33	94.44	77.78	94.44	94.44	88.89	72.22	77.78	77.78	77.78	77.78	94.44	88.89
PPV [%]	82.98	80.85	93.18	97.78	91.3	97.78	97.78	95.45	89.8	90.91	91.11	90.91	91.3	97.67	95.24
FAR [%]	11.36	13.64	6.82	0	4.55	0	0	4.55	0	9.09	6.82	9.09	4.55	4.55	9.09
LT [s]	0.007	0.004	0.005	0.005	0.004	0.004	0.004	0.004	0.004	0.004	0.004	0.004	0.004	0.004	0.004
DDT	2	3	2	1	1	1	1	2	3	3	3	2	3	2	2

**Table 2:** Result of classification based on 220 samples of signal and 4th polynomial degree for other classifiers.

	Accuracy [%]	Sensitivity [%]	Specificity [%]	PPV [%]	FAR [%]
**SDT**	**98.39**	**100**	**94.44**	**97.78**	**0**
BaggedTrees	98.39	100	94.44	97.78	0
kNN	98.39	100	94.44	97.78	0
SVM	98.39	100	94.44	97.78	0
LDA	96.77	100	88.89	95.65	0
LR	95.16	97.73	88.89	95.65	2.27
MLP	95.16	95.45	94.44	97.67	4.55
QDA	88.71	88.64	88.89	95.12	11.36
NB	87.1	86.36	88.89	95.0	13.64
